# PIM-1 kinase interacts with the DNA binding domain of the vitamin D receptor: a further kinase implicated in 1,25-(OH)_2_D_3_ signaling

**DOI:** 10.1186/1471-2199-13-18

**Published:** 2012-06-21

**Authors:** Christina J Maier, Richard H Maier, Raphaela Rid, Andrea Trost, Harald Hundsberger, Andreas Eger, Helmut Hintner, Johann W Bauer, Kamil Onder

**Affiliations:** 1 Division of Molecular Dermatology, Department of Dermatology, Paracelsus Medical University, Salzburg, Austria; 2 IMC Fachhochschule Krems, Krems, Austria; 3 Department of Ophthalmology and Optometry, SALK/Paracelsus Medical University, Salzburg, Austria

**Keywords:** Coactivator, PIM-1 kinase, Protein-Protein interaction, Serine/Threonine kinase, Vitamin D, Vitamin D receptor

## Abstract

**Background:**

The vitamin D3 receptor (VDR) is responsible for mediating the pleiotropic and, in part, cell-type-specific effects of 1,25-dihydroxyvitamin D3 (calcitriol) on the cardiovascular and the muscle system, on the bone development and maintenance, mineral homeostasis, cell proliferation, cell differentiation, vitamin D metabolism, and immune response modulation.

**Results:**

Based on data obtained from genome-wide yeast two-hybrid screenings, domain mapping studies, intracellular co-localization approaches as well as reporter transcription assay measurements, we show here that the C-terminus of human PIM-1 kinase isoform2 (amino acid residues 135–313), a serine/threonine kinase of the calcium/calmodulin-regulated kinase family, directly interacts with VDR through the receptor’s DNA-binding domain. We further demonstrate that PIM-1 modulates calcitriol signaling in HaCaT keratinocytes by enhancing both endogenous calcitriol response gene transcription (osteopontin) and an extrachromosomal DR3 reporter response.

**Conclusion:**

These results, taken together with previous reports of involvement of kinase pathways in VDR transactivation, underscore the biological relevance of this novel protein-protein interaction.

## Background

Genomic control of primary calcitriol-responsive genes is primarily achieved by binding of (phosphorylated) vitamin D receptor (VDR)-retinoid X receptor (RXR) heterodimers in a non-symmetrical head to tail arrangement to vitamin D response elements (VDRE) located within the promotor regions of target genes [1]. Corepressor proteins such as hairless, Alien, NCoR (nuclear receptor corepressor) or SMRT (silencing mediator for retinoic acid receptor and thyroid hormone receptor) link non-liganded DNA-bound VDR or antagonist-destabilized nuclear hormone receptor (NHR)-coactivator complexes to enzymes with histone deacetylase (HDAC) activity and as a result promote condensation of chromatin to repress basal transcription [2,3]. The binding of calcitriol or one of its agonists triggers the release of corepressors, accompanied by subsequent recruitment of coactivators of the p160 family and finally of more general cofactors such as CBP/p300 to mediate local chromatin remodeling and stable assembly of the basal transcriptional machinery [1]. Secondly, calcitriol can also mediate ‘rapid responses’ that are elicited by still debatable receptor(s) located near or associated with the plasma membrane and its caveolae compartments [4]. These effects occur within minutes after hormone administration, are too rapid to involve changes in gene expression but instead direct an increase in numerous second messengers that can, though, ultimately affect transcription through secondary cross-talk with other (kinase) signal transduction cascades [5,6].

Despite an already substantial quantity of literature on VDR biology - for example, 1α,25- dihydroxyvitamin D3 signaling; classification of agonistic/antagonistic VDR ligands synthesized with the goal to improve the biological profile of the natural hormone for therapeutic application; descriptions of associations between VDR and a variety of interactors as listed in the Primos (http://primos.fh-hagenberg.at) and Biogrid (http://www.thebiogrid.org) database; as well as descriptions of structural motifs and conformational changes responsible for transcriptional control - there remain important regulatory aspects and mechanisms that have not been comprehensively elucidated.

By performing a genome-wide yeast two-hybrid (Y2H) screen using human VDR as bait in order to detect new protein-protein interactions (PPIs) to obtain a more complete understanding of the mode of action of VDR, we were able to identify a previously unknown binding partner, PIM-1 (NCBI accession no. NM_002648). PIM-1 (proviral insertion of Moloney murine leukemia virus), which is a serine/threonine kinase of the calcium/calmodulin-regulated kinase (CAMK) family, phosphorylates different downstream effectors and has accordingly been proposed to play a key role in cell-cycle progression, cell survival, and differentiation of a variety of cell types [7-10]. Here we identify PIM-1 kinase as a novel VDR interacting protein whose overexpression significantly enhances and knock-down suppresses both, endogenous calcitriol response gene transcription (osteopontin) and an extrachromosomal DR3 (direct repeat of 2 hexamers spaced by 3 nucleotides) reporter response. These findings suggest involvement of the VDR-PIM-1 interaction in calcitriol-mediated growth and especially differentiation of keratinocytes.

## Results

### Genome-wide screening of a human cDNA library for VDR interacting proteins delivered PIM-1 kinase as candidate interactor

To identify novel interactors of human VDR we carried out a Y2H assay by screening full length human VDR against a bone marrow cDNA library contained in the pAD-Gate1 to 3 vector system, as described by Maier *et al.*[11]. From approximately 5 × 10^6^ yeast transformants we recovered a total of 43 different positive clones expressing putative VDR interactors. We concentrated our efforts on one clone encoding a carboxy-terminal fragment of PIM-1 kinase [amino acids (AA) 135–313], as determined via homology searches using NCBI BLAST. However, prior to further experimentation we first demonstrated the existence of PIM-1 mRNA in HaCaT keratinocytes by using gene-specific real-time PCR primers (data not shown); this positive result indicated that our newly identified protein-protein interaction could plausibly take place not only in human bone-marrow but also in human skin cells.

### The VDR DNA-binding domain is a PIM-1 interaction site

**Figure 1:**
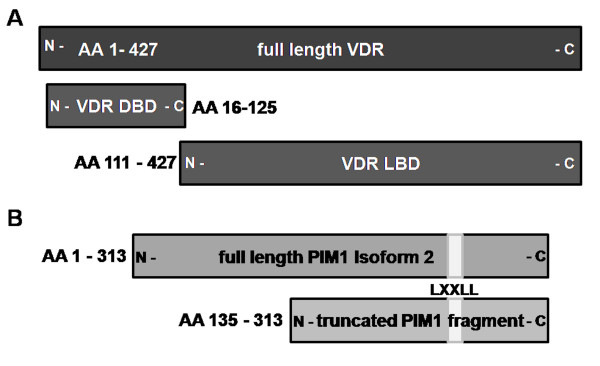
**Illustration of the used VDR/Pim-1 variants.** (**A**) In addition to full-length VDR the DNA Binding Domain (DBD: AA 16–125) and the Ligand Binding Domain (LBD: AA 111–427) of the VDR were subjected for Y2H analysis as bait molecules. They were tested with (**B**) full-length PIM-1 isoform 2 as well as the truncated version of PIM-1 (AA 135–313).

**Figure 2:**
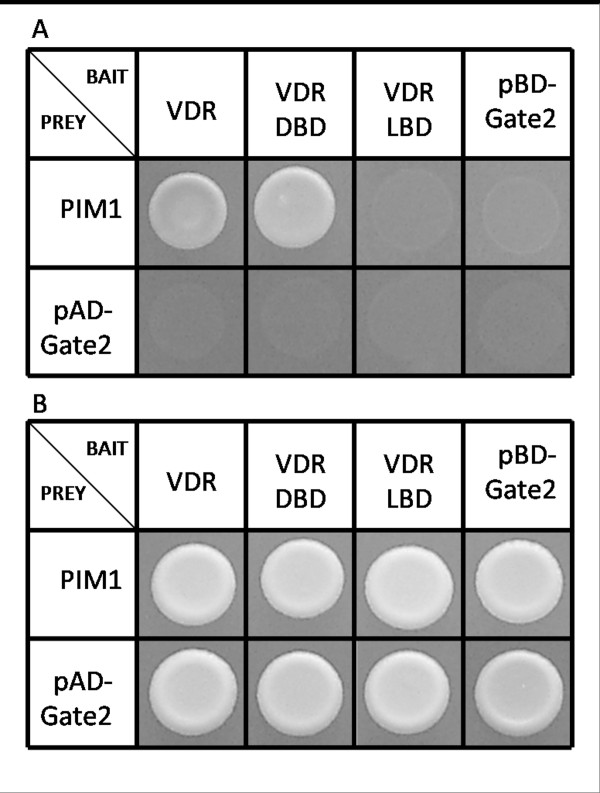
**Domain analysis of PIM-1 – VDR interaction.** (**A**) Yeast cells co-transformed with PIM-1-pAD-Gate2 and VDR-Fullength-pBD-Gate2 grow directly on PPI selective medium as well as yeast cells co-transformed with PIM-1-pAD-Gate2 and DBD-pBD-Gate2. Cells co-transformed with PIM-1-pAD-Gate2 and LBD-pBD-Gate2, PIM-1-pAD-Gate2 and pBD-Gate2, pAD-Gate2 and VDR-Fullength-pBD-Gate2, pAD-Gate2 and DBD-pBD-Gate2, or pAD-Gate2 and LBD-pBD-Gate2 show no growth on PPI selective medium. (**B**) All combination show strong growth on medium selective for positive transformation.

We isolated full-length human PIM-1 kinase from the cDNA library using gateway-compatible oligonucleotides and then subcloned the cDNA into pAD-Gate2 via standard BP-/LR-reactions. Now, using the full-length PIM-1 clone, we repeated the Y2H test against VDR and confirmed the positive interaction. To determine the VDR regions potentially involved in PIM-1 binding, we subcloned the VDR fragment encoding the DNA-binding domain and the fragment encoding the ligand-binding domain (LBD) separately into the vector pBD-Gate2 for Y2H screening against full-length PIM-1 as well as the truncated PIM-1 or appropriate negative controls (Figure 1). Yeast cells co-transformed with the VDR LBD and either full-length PIM-1 isoform 2 or truncated PIM-1 (AA 135–312) failed to grow on SD/-trp-leu-ade-his medium, whereas cells expressing both the VDR DNA-binding domain and PIM-1 kinase did grow (Figure 2). This result indicates that the PIM-1 binding site of VDR maps to the fragment encoding the VDR DNA-binding domain.

*In silico* examination of human PIM-1 kinase revealed the presence of a putative nuclear hormone receptor coregularoty LxxLL motif, namely L_228_-G-I-L_231_-L_232_ (albeit not in a conserved context), within the minimal VDR interaction domain. The general trend for LxxLL motifs (where L is leucine and x is any amino acid) that mediate the association between cofactors and liganded nuclear hormone receptors to modulate transcription includes a hydrophobic residue positioned at −1 and −7, a proline residue at −2, an aromatic residue at −3 and glutamate at location −5 to create an L-x-E/H-x-H/F-P-L/M/I-L-x-x-L-L consensus pattern [12]; however, the PIM-1 sequence in this stretch is R-S-A-A-V-W-S-L_228_-G-I-L_231_-L_232_. Although the AA residues flanking the LxxLL main core motif are known to be important for the recognition of selected classes of nuclear hormone receptors, the exact number and composition of LxxLL motifs can vary considerably among different coactivators [13].

### PIM-1 kinase and VDR colocalize in the nucleus of HaCaT keratinocytes

Mammalian expression constructs for YFP-VDR and red fluorescent PIM-1 were used to transfect cultured HaCaT keratinocytes to visualize the subcellular localization of VDR and PIM-1, and to evaluate the influence of 200 nM calcitriol (or ethanol as vehicle) on this distribution. YFP-tagged VDR was observed to be predominantly located in the nucleus independent of calcitriol treatment and residually in the cytoplasm. This result differs from previous studies, which reported that unliganded VDR partitions consistently between the cytoplasm and the nucleus, as observed in a variety of cell types such as COS-7 cells deficient in expressing endogenous VDR, CV-1 fibroblasts, rat ROS17/2.8 osteosarcoma cells, mouse adenocarcinoma cells, 293 adenovirus-transformed human embryonal kidney cells, and human dermal fibroblasts, and undergoes substantial translocation into the nucleus upon calcitriol treatment [14]. Real-time PCR experiments analyzing expression of CYP24A1 or OPN, which are well known VDR-target genes as well as Western blots of lysates of untransfected HaCaT cells in comparison to YFP-VDR-overexpressing samples (data not shown) have in this context confirmed that the tagged nuclear hormone receptor is fully functional.

**Figure 3:**
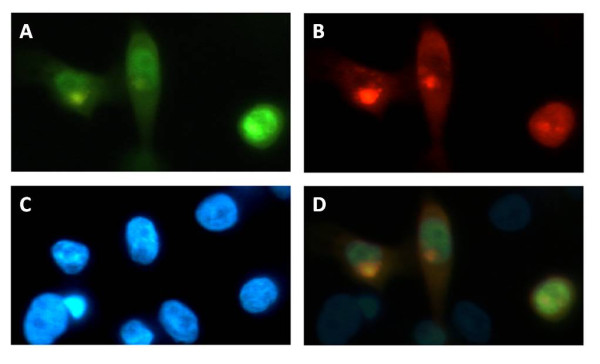
**PIM-1 and VDR co-localization in human keratinocytes.** HaCaT cells were co-transfected with PIM-1-DsRed and VDR-YFP fusion constructs. (**A**) Image of the YFP-fluorescence of VDR. (**B**) Image of the red fluorescence of PIM-1. (**C**) Image of blue DAPI fluorescence. (**D**) Overlay of panels A, B and C showing colocalization of PIM-1 and VDR in the nucleus.

PIM-1 kinase also was observed to be predominantly nuclear, however, not throughout the complete nucleus. A dense concentration of PIM-1 in an undefined local nuclear region (not corresponding to the nucleolus) was observed, where it patially colocalizes with VDR in HaCaT keratinocytes (Figure 3). A residual cytoplasmic staining was also observed. Its intracellular distribution was not obviously influenced by the presence or absence of calcitriol (data not shown). Previous studies have described both cytoplasmic and especially nuclear PIM-1 localization, as visualized via biochemical cell fractionation studies [15], transfection of a GFP-tagged PIM-1 kinase into HeLa cells [16], or anti-PIM-1 antibody staining approaches [17]; in the latter study the kinase was detected in both the cytoplasm and nucleus at an early stage of U937 PMA (phorbol myristate acetate)-induced cell differentiation but then underwent a dramatic shift toward the nucleus after 24 h of exposure to this substance.

### PIM-1 kinase is not a classical calcitriol target gene

**Figure 4:**
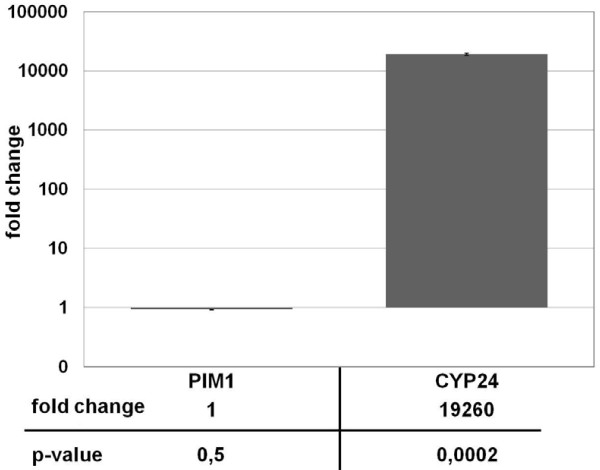
**Real-Time analysis of PIM-1 trancription in human keratinocytes upon calcitriol stimulation.** HaCAT cells were induced either with 0.2 μM calcitriol or with an equal amount of ethanol for 16 h. Levels of gene expression were analyzed by Real-Time PCR, and are shown as fold change differences between vehicle and calcitriol induction relative to the housekeeping gene ANXA1. The expression levels of PIM-1 show no significant differences between calcitriol and ethanol induction (p-value = 0,5), whereas CYP24A1 gene expression shows an enormous increase with calcitriol induction (p-value = 0.0002). Error bars represent the standard error of the mean (SEM) of n = 3 values.

To further evaluate the behaviour of PIM-1 kinase upon the treatment with calcitriol, total RNA extracted from cultured HaCaT keratinocytes as well as 293HEK (human embryonic kidney) cells that had been exposed to 200 nM calcitriol (or an equal amount of ethanol) for 16 h was reverse transcribed into cDNA and subjected to real-time PCR analysis. The RNA expression levels were observed with gene-specific primers chosen according to the published sequences of human PIM-1, CYP24A1 (encoding 25-hydroxyvitamin D24-hydroxylase or 24-OHase), a typical positive control target gene whose perceived upregulations were in accordance to recent publications [18], and ANXA1 as internal reference gene. Melting curve examinations, agarose gel electrophoresis and DNA-sequencing determined that the amplified products were indeed specific; in addition, a negative control for each RNA specimen was prepared. Following normalization, calcitriol induction in both cell types was evident, but no difference in the mRNA levels of PIM-1 kinase could be observed, suggesting that PIM-1 kinase is not a calcitriol target gene (Figure 4).

### PIM-1 overexpression enhances mRNA transcription of VDR-target genes and activates response of extrachromosomal VDR-reporter constructs calcitriol independently

**Figure 5:**
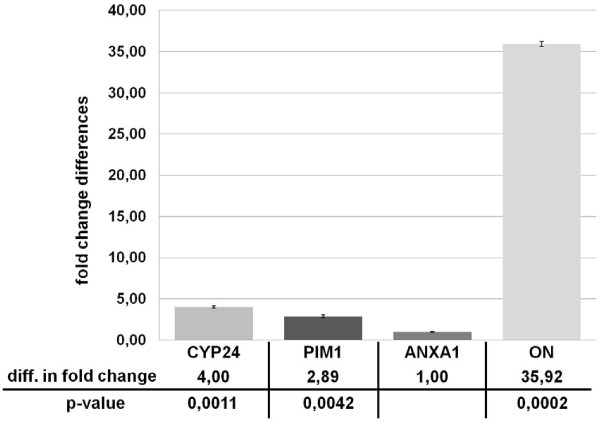
**Endogenous VDR target gene mRNA expression in PIM-1 overexpressing human keratinocytes.** With PIM-1 overexpression the levels of CYP24A1, PIM-1, and OPN are increased after normalization with the housekeeping gene ANXA1. Results are shown as fold change of PIM-1 overexpression relative to cells transfected with the empty vector. The significant differences of PIM-1 overexpression compared to the empty control vector samples was considered as true by the low p-values. Error bars represent the SEM of n = 3 values.

**Figure 6:**
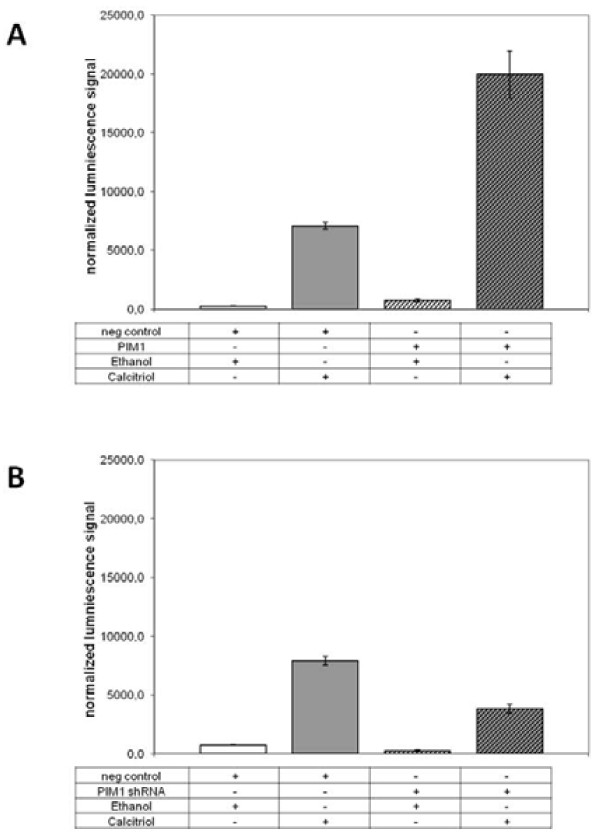
**PIM-1 dependent VDR-transactivation.** (**A**) PIM-1 overexpression enhances calcitriol-induced extrachromosomal DR3 reporter response. The luminescence signal increased after induction of the transfected HaCaT cells with 0.2 μM calcitriol for 16 h but not following the addition of an equal amount of ethanol for the same time. The cells transfected with PIM-1 showed a 2.8-fold increase in response to calcitriol compared with the cells transfected with empty vector (pDEST26/neg control). Also, similar signal intensities were observed for vehicle-treated cells transfected with PIM-1 *vs.* vehicle-treated cells transfected with the empty vector. (**B**) PIM-1 knockdown through shRNA plasmid constructs reduces the calcitriol-induced extrachromosomal DR3 reporter response to the half compared with the cells transfected with the neg. control shRNA plasmid. Furthermore, about the similar decrease was observed by calcitriol-independent extrachromosomal DR3 reporter response. Vehicle-treated cells transfected with shRNA against PIM-1 *vs.* vehicle-treated cells transfected with the empty vector (pGFP-V-RS/neg. control) reduces the signal intensity. Error bars represent the SEM of n = 3 values.

In a further real-time PCR experiment, we analyzed the effect of overexpression of PIM-1 kinase on osteopontin (OPN) and CYP24A1 (CYP24) mRNA levels in the absence of calcitriol treatment; the expression level of ANXA1 was used as an internal control. The results, presented in Figure 5, clearly show that both OPN and CYP24 mRNA levels are increased by PIM-1 overexpression (36- and 4-fold, respectively), suggesting that PIM-1 kinase overexpression provokes an calcitriol-independent enhancement of OPN and CYP24 transcription mediated by VDR. Because it is conceivable that the endogenous activation of transcription we observed is mediated through epigenetic mechanisms, we also tested a commercially available extrachromosomal DR3 reporter construct designed for accurate, sensitive and quantitative assessment of the activation of signal transduction pathways, bears no other interaction sites for additional transcription factors, and hence confirms that a direct stimulation through VDR binding takes place. For this purpose we co-transfected PIM-1 kinase construct with an equal amount of a VDRE-luc reporter construct into HaCaT cells, treated half of the samples with calcitriol and then performed a Dual-Luciferase® Reporter Assay in which we detected firefly as well as Renilla luciferase signals with a luminometer. Following normalization of the obtained firefly values by the recorded Renilla signals, we observed a greatly increased calcitriol-stimulated luciferase signal of about 2.8-fold in PIM-1-overexpressing samples compared to cells transfected with an empty expression vector (Figure 6A). Because the cells treated with ethanol showed the same enhancement in the luciferase signal compared to calcitriol induction, we conclude from these studies that overexpression of PIM-1 in HaCaT cells is able to enhance both the calcitriol-independent and calcitriol-dependent extrachromosomal DR3 reporter response.

### PIM-1 dependence of VDR mediated transcriptional response was shown by knockdown of endogenous PIM-1

To show PIM-1 dependence of VDR-promoter binding and response gene activation, we knocked down PIM-1 expression by RNAi and analysed transcriptional activation of extrachromosomal DR3 reporter construct. Therefore PIM-1 specific short hairpin RNA (shRNA) encoding plasmids were co-transfected with an equal amount of a VDRE-luc reporter construct into HaCaT cells. The transfected cells were treated half with calcitriol and then a Dual-Luciferase® Reporter Assay was performed. The knockdown of endogenous PIM-1 results in a reduced calcitriol-stimulated luciferase signal to the half compared to the control transfection (Figure 6B). The fact that in calcitriol-independent signalling the shRNA construct against PIM-1 shows about the same reduction compared to the negative control support the theory that PIM-1 is able to enhance extrachromosomal DR3 reporter response independ of calcitriol.

### PIM-1 kinase was found to take part in the VDR-interacting protein complex of human cells

To verify the interaction between PIM-1 and VDR a pull-down of DRIP (VDR interacting protein) complex from cell lysates was performed.

**Figure 7:**
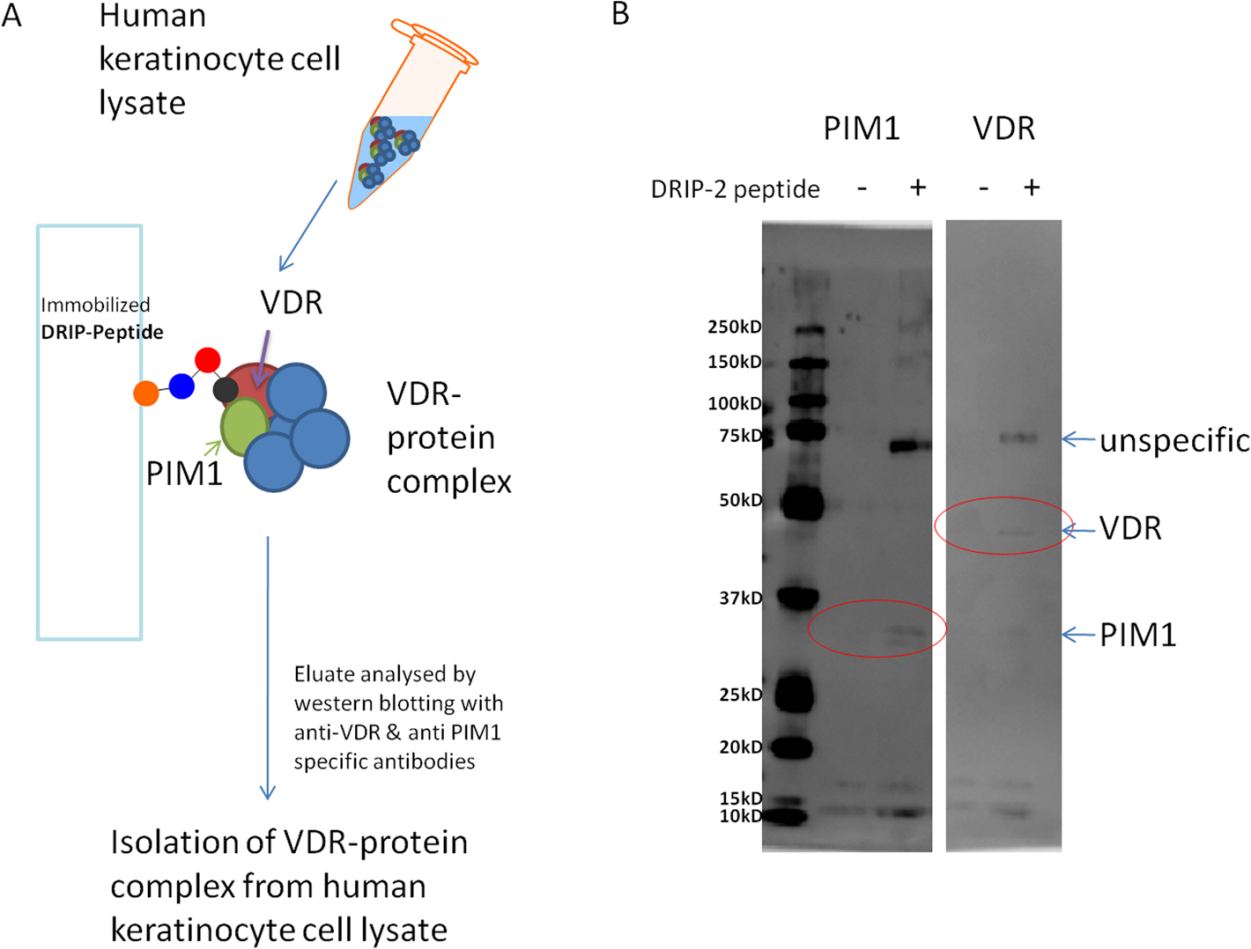
**Isolation of VDR-protein complex from human keratinocyte cell lysate.** (**A**) Overview of the experimental procedure of the pull-down assay. (**B**) Western blot analysis with anti-PIM1 and anti-VDR antibody. PIM-1 and VDR were observed only in the lysate captured by the DRIP-2 peptide, while in the control lysate without peptide only unspecific lanes appeared. The expected sizes were for VDR about 48 kD, for PIM-1 about 34 kD and for the putative VDR/PIM-1 complex (unspecific band) respectively about 82 kD.

Therefore a human keratinocyte lysate was prepared and incubated with a biotinylated synthetic VDR specific LXXLL nuclear receptor binding motif peptide, DRIP2 [19]. The biotinylated DRIP-2 peptide was immobilized using magnetic streptavidin beads. The VDR complex from human keratinocytes was captured by the immobilized DRIP-2 peptide (Figure 7). As a control for unspecific binding a lysate without DRIP-2 was incubated with the beads. The resulting eluates were analyzed by western blotting with an anti-VDR and an anti-PIM1-antibody (Figure 7).

VDR as well as PIM-1 isoform 2 could be detected in the VDR-protein complex from human keratinocyte cell lysates captured by the DRIP-2 peptide. Furthermore, an unspecific protein band appeared which is about the size of an putative VDR/PIM-1 complex (Figure 7). Despite the denaturing conditions of SDS gel electrophoresis and western blot analysis there is still the possibility of an remaining VDR/PIM-1 complex which would be a further evidence for real interaction. In the control neither VDR nor PIM-1 was detected.

## Discussion

The present study reports a previously unknown protein-protein interaction between human VDR and PIM-1 kinase and an involvement of a futher kinase in calcitriol signaling. VDR is an evolutionarily conserved member of the hormone-responsive NHR superfamily, expressed in malignant specimens (e.g. carcinomas and melanomas) and numerous mammalian tissues and, most crucially for our study, in bone and skin where it can be detected in a variety of cell types, including keratinocytes, fibroblasts, Langerhans cells, melanocytes, endothelial cells, and B- and T-lymphocytes [20,21].

PIM-1, originally identified as a locus frequently activated by proviral insertion of the Moloney murine leukemia virus [22], encodes a highly conserved 313 AA serine/threonine kinase of the calcium/calmodulin-regulated kinase (CAMK) family whose expression is stimulated by a variety of cytokines, hormones and mitogens. PIM-1 appears to be involved in the control of cell growth, differentiation, apoptosis and malignant transformation [9,15,17,23]. Putative PIM-1 kinase substrates and interactors include the c-Myb transcription factor nuclear adapter protein p100 [24], the G1-specific cell-cycle regulator Cdc25A [25], HP1 [26], the cdk inhibitor p21^cip1/waf1^[27], the nuclear mitotic apparatus protein NuMA [28] and the proapoptotic protein Bad [29], which are to a large extent involved in regulating cell-cycle progression and apoptosis. Nevertheless, the altogether small number of reported substrates would seem unlikely to be able to account for the broad spectrum of PIM-1 kinase functions, implying that it most probably interacts with additional, to date unidentified cellular substrates in specific physiological environments.

Expression of PIM-1 occurs in a wide range of tissues: hematopoietic and lymphoid tissue, prostate, testis, ovary, small intestine, colon, hippocampus and oral epithelia. It is significantly upregulated in B-cell lymphomas and erythroleukemias and has, via multi-tissue Northern blotting, been detected in a number of squamous cell carcinoma-derived keratinocyte lines, and in early and late passages of both spontaneously immortalized keratinocytes and normal human epidermal cells where its expression was substantially higher after confluence than during the logarithmic growth phase and correlated with increased keratinocyte differentiation [30-34]. There is an obvious overlap in the expression of VDR and PIM-1 kinase in a variety of calcitriol-responsive tissues.

Support for the biological significance of our discovery of a physical interaction between human VDR and PIM-1 kinase is the fact that most nuclear hormone receptors are phosphoproteins [extensively studied examples are ERα [35], PR [36], and mouse RARα [37]], whereby phosphorylation status can apparently govern receptor activity under the prevailing cellular conditions. Human VDR has been shown to be phosphorylated by protein kinase C (PKC) at serine 51 in the α-helical region that forms the two zinc fingers [38] and by protein kinase A (PKA) at serine 182 [39], which together attenuate the receptor’s VDRE-binding, RXR heterodimerization and transactivation functions. Conversely, calcitriol-stimulated, casein kinase II-catalyzed phosphorylation of human VDR at serine 208 [39-41] does not affect the receptor’s kinetics for hormone binding, its partitioning into the nucleus or its ability to associate with DNA but instead modulates its affinity for the 220 MW DRIP205/TRAP220 subunit of the large DRIP (VDR interacting proteins) mediator complex that directly anchors liganded VDR to components of the RNA polymerase II holoenzyme and hence amplifies its ability to transactivate target promoters. In addition, activated calcium/calmodulin-dependent kinase CaMKIV, which exhibits 30% identity and 45% similarity to PIM-1 (http://www.biobase.international.com), dramatically stimulates calcitriol-activated reporter gene expression by increasing VDR phosphorylation levels and results in enhanced interaction between VDR and SRC coactivator proteins in mammalian two-hybrid studies [42]. Calcitriol also activates mitogen-activated protein (MAPK) kinases in normal human keratinocytes [43] and other cell types [44], thereby providing a rapid mechanism for the hormone to promote its own receptor-mediated action and suggesting a possible connection between the well-established gene regulatory mechanisms mediated through the VDR and the rapid response cascades. Functional studies on other steroid hormone and retinoid receptors have similarly implicated phosphorylation in the regulation of DNA association [45], hormone binding [46], nuclear localization [47] and transcriptional activation [48,49]. Similar modifications have also been reported for a variety of NHR coregulators, including SRC-1, TIF2, RAC3, p300, CBP, NcoR and SMRT [50,51], which may either enhance their interaction with NHRs as well as their abilitiy to recruit histone acetyltransferase (HAT) complexes (in the case of coactivators) or induce the redistribution of corepressor from the nucleus to the cytoplasm, thereby lessening the potential association between corepressors and NHRs [52-54].

Our domain mapping approaches have nevertheless indicated that the interaction with PIM-1 kinase does not involve the ligand-binding domain of VDR in contrary to the majority of co-activators. The interaction between PIM-1 and VDR is instead restricted to the receptor’s DNA binding domain, a type of interaction that, to our knowledge, has been described only 3 times previously: (i) between VDR and the bone-specific transcription factor Runx2 as a prerequisite to upregulate rat osteocalcin expression in osteoblasts [55]; (ii) between VDR and acute promyelocytic leukemia zinc finger protein (PLZF) necessary for the regulation of calcitriol-induced monocytic differentiation in hematopoietic cells [56]; and (iii), in a different experimental context, between VDR and the oncogenic nucleoporin CAN/Nup214, a component of the nuclear pore complex [57].

Because yeast two-hybrid experiments do not allow any conclusions about protein-protein interactions in a native cellular environment, we also studied PIM-1 and VDR in the spontaneously immortalized human HaCaT cell line which is known to behave phenotypically like normal basal epidermal keratinocytes in terms of growth and differentiation and to develop in the absence of serum and supplementary exogenous growth factors, features which make it a widely used cell culture system for such studies and an important model for the highly proliferative epidermis [58]. As visualized by overexpressing YFP-VDR and tomato-tagged PIM-1, we could observe significant co-localization of the two proteins in the nucleus of HaCaT keratinocytes, which confirms that both proteins could indeed have the opportunity to interact with each other at the physiological level. We were subsequently able to increase transcription of CYP24A1 and osteopontin mRNA, the latter a classic VDR response gene [59,60], by approximately 50% upon extrachromosomal overexpression of PIM-1 kinase. Furthermore, we similarly observed a clear enhancement of extrachromosomal DR3 reporter response upon PIM-1 overespression and respectively a clear reduction in DR3 reporter response by PIM-1 shRNA mediated knock-down, suggesting that PIM-1 kinase indeed can modulate calcitriol signaling. In an earlier study, Thompson *et al.*[61] analyzed whether PIM-1 was capable of modulating androgen signaling because androgens are known to play a key role in male physiology as well as in the promotion or progression of prostate cancer and PIM-1 kinase was reported to be markedly overexpressed under such conditions [34]. Ruling out simple explanations like altered receptor levels or DNA binding characteristics, they observed that, under transient transfection conditions, PIM-1 attenuated the transcriptional activity of AR, another NHR superfamily member (and in parallel experiments also of a LBD-deleted AR, GR and ERα), in a dose-dependent fashion in several cell types, but found no evidence for direct phosphorylation of AR by PIM-1 or, in contrast to our results, for a direct protein-protein interaction [61]. They therefore suggested that PIM-1 likely influences AR activity via an indirect mechanism that possibly involves phosphorylation of a coregulator and/or a component of the transcriptional machinery. We could show that PIM-1 binds to the DNA-binding domain of VDR and modulates calcitriol signaling, however, we did not analyse the phosphorylation of VDR by PIM-1. Therefore it remains unclear whether VDR or a different component of the pathway is modulated by PIM-1. Interestingly, and in this context it was reported that PIM-1 enhances the transcriptional activity of c-myb, another transcription factor, by direct phosphorylating the DNA-binding domain as well as the phosphorylation of the transcriptional co-activator p100 [24,62].

In a final pull-down of VDR-interacting protein complexes we investigated the protein complex of VDR by using the facility of a LXXLL containing peptide (DRIP-2) to capture the VDR-interacting co-activator protein complex in cell lysates. We detected the PIM-1 isoform 2 in the eluted fraction containing also VDR, but there is no detection of endogene PIM-1 in the fraction without DRIP-2 capture, so PIM-1 indeed seems to be included into the VDR-complex captured by DRIP-2 peptide, and potentially a member of the DRIP205-VDR co-activator complex [19].

## Conclusion

Because the interaction between PIM-1 kinase and VDR involves the DNA-binding domain of the latter, it is conceivable that this association supports the binding of VDR to VDRE response elements within specific target genes, a hypothesis that will be tested in forthcoming studies aimed at clarifying the mechanism of co-activation.

## Methods

### Genome-wide Y2H screening

To construct the bait, the 1284 bp sequence encoding full-length human vitamin D receptor (NCBI accession no. NM_000376) was cloned by recombinational cloning into pDONR/Zeo (Invitrogen, Carlsbad, CA, USA), shuttled into the GAL4 DNA-binding domain (BD)-containing vector pBD-Gate2 [11], and sequenced by using an ABIPRISM^TM^ Cycle Sequencing Kit and an ABI-PRISM^TM^ 310 Genetic Analyzer (Applied Biosystems, Foster City, CA, USA). In a preliminary experiment, in which the known interaction between p53 and SV40 large T antigen [63] served as positive control, the construct was found to have no capacity to nonspecifically activate reporter genes. A commercially available human bone marrow cDNA library in the Gateway-compatible pCMV-SPORT6 vector (Invitrogen) was transferred via recombinational cloning into the GAL4 activation domain (AD) containing vectors pAD-Gate1, -Gate2, and -Gate3 [11] vectors and purified by using a GenElute HP Plasmid Maxiprep kit (Sigma-Aldrich, Taufkirchen, Germany), resulting in three libraries that differ from each other only in the translational reading frame. To perform a large-scale Y2H screening with the MATCHMAKER GAL4 Two-hybrid System 3 (Clontech, Mountain View, CA, USA), the VDR-pBD-Gate2 bait and each of the 3 libraries were simultaneously co-transformed into the haploid *Saccharomyces cerevisiae* strain AH109 (genotype: *MATa, trp1-901, leu2-3, 112, ura3-52, his3-200, gal4Δ, gal80Δ, LYS2:: GAL1*_*UAS*_*GAL1*_*TATA*_*-HIS3, GAL2*_*UAS*_*-GAL2*_*TATA*_*ADE2;URA3::MEL1*_*UAS*_*-MEL1*_*TATA*_*-lacZ MEL1*) according to a standard high-efficiency lithium acetate method, and finally plated onto (i) a nutritionally selective plate deficient in tryptophan and leucine (SD/-leu-trp) to test for positive plasmid uptake and general transformation efficiency, and (ii) SD/-trp-leu-ade-his synthetic medium to select for actual reporter activity. The plates were incubated for at least 4 days at 28 °C. The cDNA library vectors from primary positive yeast candidate clones were recovered by plasmid isolation via digestion with 10 U lyticase (Sigma-Aldrich), addition of 10% SDS, one freeze-thaw cycle, and purification by using a Wizard® SV gel and PCR clean-up system (Promega, Madison, WI, USA). The plasmids were then amplified in *E. coli* DH5α cells and reintroduced back into the yeast strain AH109 together with the bait or empty pBD-Gate2 (as a control) and finally retested on SD/-trp-leu-ade-his in order to verify their reporter activity before the sequence of the clone was determined by using vector-specific primers. Bioinformatical analyses were conducted through the National Center for Biotechnology Information (NCBI) by using the basic local alignment search tool (BLAST) accessible via http://blast.ncbi.nlm.nih.gov/Blast.cgi, the ExPASy Proteomics Server (http://www.expasy.ch/), the Universal Protein Resource UniProt (http://www.uniprot.org/) and the SWISS-PROT knowledgebase (http://expasy.org/sprot/). Only putative VDR-interacting candidates passing the control experiments were subjected to further study.

### Mapping the interacting domains

Full-length human PIM-1 kinase isoform 2 subcloned into pDONR/zeo was shuttled into the pAD-Gate2 vector via an LR reaction and used as the prey plasmid in our subsequent domain mapping experiments. The ligand-binding domain (LBD, amino acids 111–427) and the DNA-binding domain (DBD, AA 16–125) of human VDR were similarly cloned into pDONR/Zeo and subsequently shuttled into pBD-Gate2, resulting in bait proteins fused to the GAL4 DNA-binding domain. To identify the protein regions involved in the interaction between PIM-1 kinase and VDR, the particular bait and prey vectors (or the respective empty vectors as negative controls) were co-transformed into the haploid yeast strain AH109 as described above. Positive interactions were indicated by cell growth on SD/-leu-trp-ade-his medium after 4 days of incubation at 28 C. Because direct plating of transformants onto SD/-leu-trp-ade-his medium represents a rather stringent condition that selects for only strong interactors, and hence could fail to detect low-affinity, auto-activating, and perhaps transient interactions, we also tested a less stringent condition, i.e. we picked several random clones from SD/-leu-trp plates, dissolved them in water and finally dropped them onto SD/-leu-trp-ade-his [11].

### Cell culture techniques and transfection

Cell culture experiments were performed with the spontaneously immortalized human keratinocyte cell line HaCaT that was maintained as a monolayer culture under a 5% CO_2_ at 37 C in Epilife® medium (Cascade Biologics/Invitrogen GmbH, Karlsruhe, Germany) together with defined human keratinocyte growth supplement (HKGS), 60 μM CaCl_2_, and 0.5% penicillin/streptomycin. For transfection experiments, 1-3×10^5^ HaCaT cells were seeded in flasks or chamber slides that allow cell cultivation in a microscopic carrier (BD Biosciences, Erembodegem, Belgium, Nunc^TM^/Lab-TEK®, Langenselbold, Germany) to reach approximately 50% to 80% confluence, and electroporated by using a Cell line Nucleofector® kit V (Amaxa Biosystems, Lonza GmbH, Wuppertal, Germany) according to the manufacturer’s instructions with a total of 5 μg plasmid DNA.

### Co-localization studies

Human VDR subcloned into pDONR/Zeo [11] was shuttled into pcDNA^TM^6.2/N-EmGFP/YFP-DEST (Invitrogen) via a standard LR reaction to create an N- terminally yellow fluorescent protein (YFP)-tagged protein. Human PIM-1 was shuttled from the entry vector into a Gateway®-compatible mammalian red fluorescent pDEST/Tomato destination plasmid which we constructed through insertion of a tomato dimer sequence amplified from pRSET-B tdTomato (Invitrogen) into pcDNA^TM^3.1D/V5-His-TOPO (Invitrogen). To investigate the actual co-localization between PIM-1 and VDR, exponentially grown HaCaT cells transfected with a combination of YFP-VDR and Tomato-PIM-1 were cultured onto chamber slides as described above, rinsed with 1x PBS, fixed in 4% paraformaldehyde in 1x PBS for 10 min and counterstained with 1 μg/ml 1,4,6-diamidino-2-phenylindole (DAPI). After three additional washing steps, the coverslips were inverted into mounting medium (MicroTrak®, Trinity Biotech PLC, Wicklow, Ireland) and the fluorescence staining was evaluated with a Zeiss Axioskop MC100 (Carl Zeiss MicroImaging GmbH, Göttingen, Germany).

### Real-time PCR analysis

Total RNA from HaCaT keratinocytes grown to approximately 90% confluence was isolated with an RNeasy Midi Kit (Qiagen GmbH, Hilden, Germany) according to the kit’s manual. RNA purity and concentration were analysed using a Nanodrop® ND-1000 UV/Vis Spectrophotometer (PEQLAB GmbH, Erlangen, Germany) and RNA integrity visually assessed by agarose gel electrophoresis. After a 20-min digest with DNase I (Sigma-Aldrich) at 25 C to remove DNA contamination, 0.55 μg of purified RNA was reverse transcribed into cDNA with an iScript™ cDNA Synthesis Kit (Bio-Rad Laboratories, Hercules, CA, USA) by incubating the reaction for 5 min at 25 C, for 30 min at 42 C, and for an additional 5 min at 85 C in a programmable thermocycler. The resulting cDNA (or an equal amount of DNase-digested RNA for a negative control) was used as a template for quantitative real-time PCR using an iQ™ SYBR green Supermix, an iCycler™ apparatus (both Bio-Rad Laboratories) and the following conditions: a hot-start incubation at 95 C for 5 min and 50 cycles consisting of denaturation at 95 C for 30 s, primer annealing at 60 C for 20 s, and elongation at 72 C for 20 s, followed by 50 cycles of fluorescence acquisition that determines the specifity of the amplified products and verifies the absence of oligonucleotide-dimer formation. All PCRs were carried out in a final total volume of 25 μl and were performed in triplicates for each cDNA sample. The real-time primers used in this study were designed by using Primer3 software available at http://frodo.wi.mit.edu/cgi-bin/primer3/primer3_www.cgi. A complete list of oligonucleotides is given in Table 1. The obtained data were normalized against annexin A1, an unregulated endogenous reference transcript, as control for any analytic variation. Results were analyzed with the iCycler iQ™ optical system software 3.1 (Bio-Rad Laboratories, Hercules, CA, USA) by comparing the distinct cycle threshold values (c_t_). The differences between the analyzed samples compared to their controls were shown as fold change and respective p-values were calculated using a homoscedastic *t*-test (only data with p-value < 0.05 were considered statistically significant).

**Table 1:** Primer sequences for the Real-Time PCR Analysis

**gene name**	**Abbreviation**	**Acc No**		**Sequence**	**Product size**
Annexin	ANXA1	NM_000700	forward	5´- GCAGGCCTGGTTTATTGAAA −3´	203 bp
			reverse	5´- GCTGTGCATTGTTTCGCTTA −3´	
Cyp24A1	CYP24	NM_000782	forward	5´- GGCAACAGTTCTGGGTGAAT −3´	249 bp
			reverse	5´- TATTTGCGGACAATCCAACA −3´	
Osteopontin	ON	NM_000582	forward	5´- TGAAACGAGTCAGCTGGATG −3´	162 bp
			reverse	5´- TGAAATTCATGGCTGTGGAA −3´	
PIM-1 kinase	PIM1	NM_002648	forward	5´- CAGAGTGGATCCGCTACCAT −3´	226 bp
			reverse	5´- TGGATTTCTTCGAAGGTTGG −3´	

### Reporter assay measurements and PIM-1 knockdown

PIM-1 kinase was shuttled via an LR reaction into the mammalian destination vector pDEST26 (Invitrogen) and transfected into HaCaT cells together with a VDRE-luc reporter (Cignal Vitamin D Reporter Kit, CCS-2029 L, SABiosciences Corporation, Frederick, MD, USA), which gives reproducible, sensitive and specific measurements of changes in transcriptional activities. The VDRE-luc reporter is a mixture of (i) an inducible firefly (*Photinus pyralis*) luciferase reporter gene under control of a basal TATA box control element joined to tandem repeats of a specific transcriptional response element, and (ii) a constitutively expressed Renilla (*Renilla reniformis*) luciferase regulated by a CMV immediate early enhancer/promoter that can be used as an internal reference for standardizing experimental variabilities such as transfection efficiencies and cell viability. As a negative control, empty pDEST26 was co-transfected with VDRE-luc in a parallel approach. Transfected samples were split into a Corning® CellBIND® Surface plate (Corning Life Sciences, Lowell, MA, USA) and incubated for 48 h at 37 C before the samples were treated with 0.2 μM calcitriol or an equal amount of ethanol as vehicle for further 16 h. The cells were then gently and rapidly lysed with passive lysis buffer (provided with the kit), which is formulated to circumvent the need for scraping of adherent cells or additional freeze-thaw cycles. The assay was developed by measuring the activities of firefly and Renilla luciferase reporters sequentially from each sample by using a Dual-Luciferase® Reporter (DLR™) System (Promega). Promoter activity values were expressed as arbitrary units after normalization and correction for background reactivity. The PARADIGM™ Detection Platform (Beckman Coulter® Inc., Fullerton, CA, USA) was used for signal detection.

For PIM-1 knockdown PIM-1 specific short hairpin RNA (shRNA) encoding plasmid constructs in pGFP-V-RS was obtained from Origene (HuSH™-29; Origene, Rockville, MD, USA). The set of four 29mer shRNA constructs (Table 2) were co-transfected with VDRE-luc reporters into HaCaT cells. As negative control the empty shRNA pGFP-V-RS vector (Origene) was used with VDRE-luc. The assays were induced by calcitriol or an equal amount of ethanol and measured in the same way. All data between calcitriol treated and control cells were calculated to be statistically significant using a homoscedastic *t*-test (only data with p-value < 0.05 were considered statistically significant).

**Table 2:** PIM-1-specific shRNA sequences for PIM-1-knockdown

**construct**	**29mer Sequence**
GI362006	TGG AAG TGG TCC TGC TGA AGA AGG TGA GC
GI362007	CTG CTC AAG GAC ACC GTC TAC ACG GAC TT
GI362008	CCT GAG ACC ATC AGA TAG GCC AAC CTT CG
GI362009	CAG AAT GTC AGC ATC TCA TTA GAT GGT GC

### Isolation of VDR-protein complex from human keratinocyte cell lysate

HaCaT keratinocytes grown to approximately 90% confluence were lysed using RIPA-buffer (Sigma-Aldrich,Taufkirchen, Germany). The flasks were washed twice with 10 ml cold PBS, incubated in 750 μl RIPA-buffer with protease inhibitor cocktail for 10 min at 25 °C followed by 10 min incubation at −80 °C. Cells were collected with a cell scrapper, and quantified using a classical Bradford assay.

6 mg of the lysates were pre-incubated with 50 μl magnetic Streptavidin beads (Dynabeads®MyOne™Streptavidin T1 Invitrogen) for 10 min. The beads were captured and the lysates were incubated without and with 1 μg of a synthetic biotinylated DRIP-2 peptide (Genway Biotech.Inc, San Diego, CA, USA) overnight at 4 °C with gentle agitation. The DRIP-2 peptide is an LXXLL motif containing peptide (N – NTKNHPMLMNLLKDNPAQD – C) with the facility to bind the VDR [19].

After overnight incubation 100 μl magnetic streptavidin beads were added to the lysates and incubated for 1.5 h at 25 °C. The beads were washed 4 times each 10 min with TBS + 0.05%TWEEN and then captured by a magnetic stand. The beads were resolved with 30 μl LDS-loading buffer and incubated for 5 min at 96 °C to elute the complex. 5 μl of each eluted protein peptide complexes were further analyzed by standard western blot procedures. The primary antibodies were the commercially available rabbit polyclonal IgG anti-human VDR antibody (sc-1008; Santa Cruz Biotechnology, Santa Cruz, CA, USA) for the VDR and the commercial available rabbit polyclonal IgG anti-human PIM-1 antibody (sc-28777; Santa Cruz Biotechnology) for PIM-1. For detection, an HRP conjugated swine anti-rabbit polyclonal IgG antibody (P0217; Dako, Glostrub, Denmark) was used.

## Competing interests

The authors declare that they have no competing interests.

## Authors’ contributions

KÖ provided the original concept of the study, supervised the study and contributed to writing the paper. CJM and RHM performed all working steps, were responsible for the data analysis and the manuscript preparation. RR, AT and AE provided comments and revisions to the manuscript. HaHu contributed to all biochemical experiments and supported to VDR protein related parts of the study. JWB and HeHi gave scientific support to the manuscript. All authors read and approved the manuscript.
